# SynTView — an interactive multi-view genome browser for next-generation comparative microorganism genomics

**DOI:** 10.1186/1471-2105-14-277

**Published:** 2013-09-22

**Authors:** Pierre Lechat, Erika Souche, Ivan Moszer

**Affiliations:** 1 Institut Pasteur, Plate-forme Bioanalyse Génomique, 28 rue du Docteur Roux, Paris, Cedex 15 75724, France; 2 Current address: Laboratory for Molecular Diagnosis, KU Leuven, O&N I Herestraat 49, B-3000, Leuven, Belgium

**Keywords:** Genome browser, Microbial genomics, Synteny, Next-Generation Sequencing (NGS), Single Nucleotide Polymorphism (SNP), Flash, Interactive graphical user interface

## Abstract

**Background:**

Dynamic visualisation interfaces are required to explore the multiple microbial genome data now available, especially those obtained by high-throughput sequencing — *a*.*k*.*a*. “Next-Generation Sequencing” (NGS) — technologies; they would also be useful for “standard” annotated genomes whose chromosome organizations may be compared. Although various software systems are available, few offer an optimal combination of feature-rich capabilities, non-static user interfaces and multi-genome data handling.

**Results:**

We developed SynTView, a comparative and interactive viewer for microbial genomes, designed to run as either a web-based tool (Flash technology) or a desktop application (AIR environment). The basis of the program is a generic genome browser with sub-maps holding information about genomic objects (annotations). The software is characterised by the presentation of syntenic organisations of microbial genomes and the visualisation of polymorphism data (typically Single Nucleotide Polymorphisms — SNPs) along these genomes; these features are accessible to the user in an integrated way. A variety of specialised views are available and are all dynamically inter-connected (including linear and circular multi-genome representations, dot plots, phylogenetic profiles, SNP density maps, and more). SynTView is not linked to any particular database, allowing the user to plug his own data into the system seamlessly, and use external web services for added functionalities. SynTView has now been used in several genome sequencing projects to help biologists make sense out of huge data sets.

**Conclusions:**

The most important assets of SynTView are: (i) the interactivity due to the Flash technology; (ii) the capabilities for dynamic interaction between many specialised views; and (iii) the flexibility allowing various user data sets to be integrated. It can thus be used to investigate massive amounts of information efficiently at the chromosome level. This innovative approach to data exploration could not be achieved with most existing genome browsers, which are more static and/or do not offer multiple views of multiple genomes. Documentation, tutorials and demonstration sites are available at the URL: http://genopole.pasteur.fr/SynTView.

## Background

Over the past five years, developments in the technology for determining DNA sequences have led to a substantial change in genome sequencing strategies. Applications in microbial genomics range from re-sequencing collections of strains to non-coding RNA identification, transcription start site determination, and extended metagenome studies [1]. Current high-throughput sequencing devices — *a*.*k*.*a*. “Next-Generation Sequencing” (NGS) technology — can yield hundreds of gigabases of sequence data rapidly and at an ever-decreasing cost. However, the ratio between the production of these sequence data and computer storage capacities is steadily increasing, as is the cost of data management and analysis [2]. As a consequence, the bottleneck affecting large-scale genomics projects is shifting from data generation to data interpretation. Downstream from the primary and secondary levels of analysis — such as quality control, read mapping and *de novo* assembly — the most time-consuming and specialised tool-demanding tasks are now related to data mining and exploration. To facilitate this analysis step, flexible visualisation interfaces are required [3].

Comparative genomics stands out as one of the major applications of high-throughput sequencing. In the field of microbiology, this discipline involves the elucidation of genetic traits underlying specific (non-)pathogenic phenotypes, and contributes to deciphering evolutionary relationships within large collections of isolates, with direct clinical applications [4,5]. The most frequent strategy for comparing genome organisations is to examine synteny relationships between genomes, which is the association of gene similarity with gene ordering along the chromosomes. These data can be usefully complemented by information about polymorphism at the nucleotide level; obtained at the genome scale by NGS analyses, biodiversity data help to reveal global trends in evolutionary relationships, and structural variation events such as recombination or insertion/deletion of genomic islands.

A large number of software systems have been developed in the last decade to facilitate comparative genomic studies; they give access to diverse features, in terms of type and quantity of data, analysis methods and styles of user interfaces [6-29] (and see the Discussion below). Many are web-based applications, which tends to hinder interactivity as standard HTML does not enable a dynamic user experience. Some of these tools are more user-friendly, either because they use advanced web protocols (*e*.*g*., HTML5) or because they are based on native desktop frameworks; however, these applications are mostly limited to the visualisation of one single or few genomes and/or to limited and particular types of data representation.

Here, we present a novel software application — SynTView — providing the user with substantial flexibility for comparative analysis of microbial genome information. Its originality stems from the combination of many features individually available in other applications: it is a web-based and desktop-capable Flash software; it offers highly interactive graphical user interfaces; it is designed for the visualisation, through many complementary perspectives, of both synteny relationships and DNA polymorphism data for multiple bacterial genomes. The SynTView software is therefore ideally suited to the exploration of data obtained in NGS-based population genomic studies.

## Implementation

The SynTView software was written in ActionScript 3 (AS3) language, using the Apache Software Foundation Flex framework [30] and the associated plug-in for the Eclipse™ Integrated Development Environment (IDE). This technology allows Adobe® Flash software to be built, providing dynamic presentations of complex data through either web browsers or desktop clients. Some interactive data visualisations were implemented using the Flare library [31].

SynTView was built around the following rationale: *n* genomes should be compared with *one reference* genome (*n* can be as large as the computer memory will allow). Depending on the mode of access to the application (see below), genome data can be loaded either by the user using import functions (desktop client) or with the help of a software administrator (web configuration — the only requirement is this case is to place appropriately formatted flat files in the right directory). These data can include complete annotated genomes and associated comparison files for synteny visualisation, or polymorphism data from NGS data sets. Currently, the accepted file formats are ptt (GenBank) for genome annotation, tab-delimited files for other information (protein correspondence, Single Nucleotide Polymorphisms — SNPs) and the Newick format for phylogenetic trees.

The user can access the application in three different ways. First, a stand-alone client can be run using the Adobe desktop application cross-platform runtime AIR (Adobe Integrated Runtime), which will be installed automatically along with SynTView, if required. This allows the users to work with their own locally stored data. Alternatively, web access for multiple users can be implemented; in this configuration, SynTView uses flat files stored on the web server. Finally, SynTView is available in combination with the GenoList integrated environment [32], a multi-genome microbial database accessible on the web, which houses 750 bacterial genomes [33]. This option can exploit the comparative genome data pre-computed in GenoList.

Extensive performance tests have shown that the application reached technological limits if more than ten thousand dynamic graphical objects (*i*.*e*., selectable and scalable objects) were used. Drawing strategies were thus developed to ensure that graphical outputs remain below this upper limit, using a mix of static and dynamic objects. For instance, SNP local maps come up with three display modes according to the density of polymorphic positions and the representation scale: (i) in the local view, SNP graphical objects remain interactive (*i*.*e*., presence of associated contextual menus, animation of the related gene, etc.) when the genome region displayed is not longer than 200 kb; (ii) if the region is longer than 200 kb, a static drawing is computed; and (iii) SNPs can also be represented as blocks that cluster a series of adjacent polymorphic positions together (the number of SNPs per kb is indicated by the colour of the block) — these blocks are clickable to return to the interactive local view.

## Results

### A generic genome browser

The SynTView software is built as a generic genome browser including sub-maps that hold information about genomic objects (annotations, in the broad sense). After selecting genomes of interest, users can explore them visually by genomic location, or directly go to specific genes by name (auto-completion feature). Several genomic maps can be stacked on top of each other. The user can dynamically change their respective order, adjust the scale of the maps to use the full area of the screen, and zoom in and out. Organisms can be ordered according to their evolutionary relationships using a phylogenetic tree, whose branches can be graphically flipped. The user can also browse the pivot genome or the content of gene baskets through gene tables, with sorting functionalities and backward access to the graphical view.

Contextual menus are associated with genes, allowing the user to compute local views around any particular gene, to get sequence information, to go to the GenoList gene card (if SynTView is used in its GenoList-embedded version), and to add genes to a gene basket that can subsequently be used for various operations on gene lists. More generally, cross-references from SynTView genes to other repositories offering web services (format prefix-URL + Identifier) can be implemented, as long as shared identifiers can be defined. In addition, web services can be connected to the application to perform various operations on biological objects or their relationships (*e*.*g*., multiple alignments as described in the next section). Symmetrically, external applications can control SynTView: calling methods can be specified from their own pages that will trigger the generation of a specific SynTView representation (*e*.*g*., to centre the view around a particular gene).

**Figure 1:**
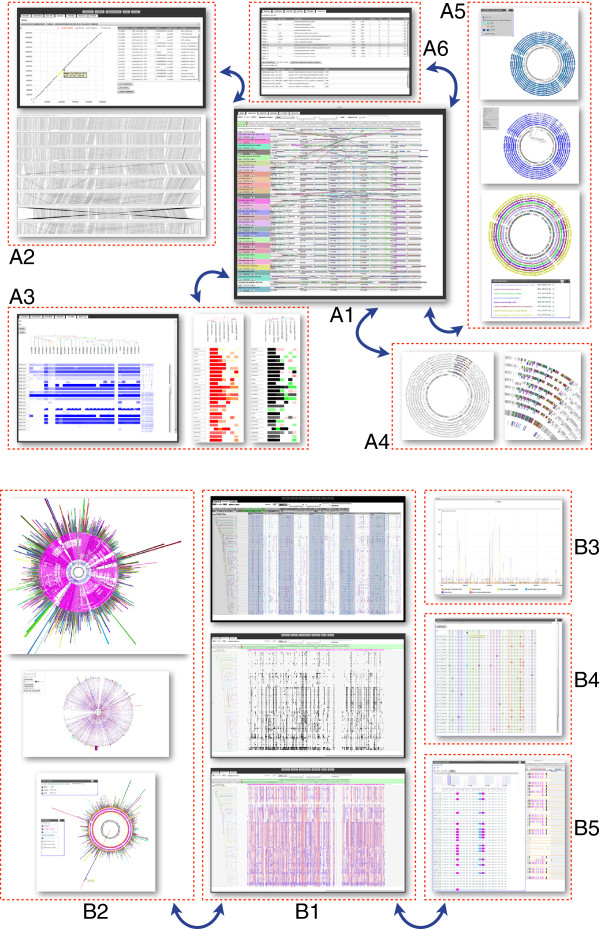
**Multiple connected views of the SynTView graphical user interface.** Panel **A** shows several representations of genome syntenic organisations and protein similarity relationships. A1: Central view showing a genome browser with dynamic links between one reference genome (top) and several synteny-aligned related genomes; A2: Dot plot (with a connected gene table on the right-hand side) and line plot representations; A3: Phylogenetic profiles representing either the whole reference genome (left-hand side) or a defined gene set (right-hand side); the colour and shape of the trapezoidal box represent the sequence similarity and protein coverage, respectively; A4: In contrast to the central local view, where absolute coordinates of aligned genomes do not coincide, this global circular view indicates whether the conserved syntenic region is localised identically in complete genomes; A5: Several kinds of circular representations where the outer circle colours represent either sequence similarity heat maps (top) or genome categories (bottom); A6: Gene table with selection widgets and BDBH display. Panel **B** illustrates the exploration of polymorphism information. B1: Three linear views of phylogenetically ordered genomes showing information for SNPs at various scales and in various representation modes: individual SNPs coloured according to their location in relation to genes (top), SNP clusters (middle) and SNP heat maps (bottom); B2: Several circular representations of SNP distributions in *n* genomes compared to one reference genome (centre), the outside bars indicate the cumulated number of SNPs per gene; B3: Histogram of metadata-clustered SNPs enabling a differential analysis by groups of strains; B4: Artificial sequence reconstituted with polymorphic positions; B5: Gene browser representing the effect of mutated positions on the nucleotide or protein sequence. The blue arrows represent the dynamic integration between the views.

Two specialised modules are available to explore and compare genome organisations and polymorphism data, as described below. These functions comprise a number of dedicated inter-connected graphical views: object selections in one view are automatically mirrored in another view (Figure 1). In addition, generic functions automatically adapt their behaviour to the context (*i*.*e*., synteny or polymorphism visualisation); for instance, strain ordering can be guided either by an autoscore computation or by a phylogenetic tree.

### Visualisation of synteny relationships

The software can display synteny relationships between a reference genome and a set of related genomes. The synteny information is typically computed from the correspondence between proteins of different organisms (*e*.*g*., Bi-Directional Best Hits — BDBH — from all-against-all BLASTP reports [34]) and the conservation of ordering of associated genes along the genomes. Protein associations can be either externally computed by any method yielding compatible files (see Implementation section and the software documentation) or obtained from pre-computed comparative data in GenoList.

To represent the synteny information, a colour is randomly assigned to every gene of the reference organism; the related genes in other genomes get the same colour as the reference gene whereas orphan genes remain in black. Clicking on a gene shows its local synteny: the other genomes shift into alignment with the main sequence and the non-syntenic genes fade away. Protein multiple alignment, when available, can be visualised by clicking on the links between the corresponding genes: this redirects the user either to GenoList or to an external alignment web service.

In addition to the local view, three global views are available. The dot plot shows synteny ruptures and chromosome re-arrangements; the matching genes can therefore be selected graphically and exported to a file. The line plot shows the organisation of syntenic groups at the chromosome level. The phylogenetic profile consists of a heat map of BLASTP hits sorted according to a phylogenetic tree. These views sport widgets for filtering the data display dynamically, according to criteria such as keywords, thresholds on BLAST reports, or the content of user-defined gene baskets.

### Visualisation of polymorphism information

The second specialised module of SynTView is designed to visualise information about polymorphism across a large number of bacterial strains. Such data are typically obtained by population genomics studies involving high-throughput genome (re)sequencing of a collection of isolates. SNPs are the most frequently represented data. They must be determined independently from the SynTView software, and formatted according to the documentation. We have developed our own procedures for computing such data; these procedures allow fine tuning of a large number of parameters for SNP identification, and are available together with the SynTView software.

The SNPs are computed and represented using a reference genome, to which *n* related strains are compared. The SNP maps allow the user to navigate through polymorphism data sets, in which SNPs are mutually linked to cognate genes (*e*.*g*., when the mouse cursor is over a gene, an animation of the enclosed and surrounding SNPs is triggered). The SNPs are coloured according to the type of mutation they cause (*e*.*g*., synonymous or not, or stop codon) and to their location in relation to genes (*i*.*e*., inside a gene or in an intergenic region); they can be shown/hidden dynamically according to these properties. Sequence variations (both at the nucleotide and protein levels) can be represented in a dedicated view. Finally, an artificial sequence consisting of the alleles at the SNP positions can be determined for all the genomes or for a group of genes, so as to compute phylogenetic distances. All the types of sequences (reference, mutated or artificial) can be exported.

Many additional features are available in the polymorphism module. For instance, the sequencing coverage can be displayed, and scale options can reveal heterogeneously covered areas. Strains can be grouped according to epidemiological information, phylogenetic information or other metadata to obtain SNP density maps (*i*.*e*., histograms in which the size of the bars represents the number of SNPs per gene); these metadata can also be used to arrange or represent SNP tracks in the main view differentially. Finally, the SNPs can be distinctively coloured or displayed according to their frequency of occurrence in the set of strains included in a given analysis (this yields representations analogous to heat maps, such as Figure 1 described in the article by Croucher and coll. [35]).

## Discussion

The software features described in this paper illustrate only some of the main capabilities of the SynTView application. A full list of its functionalities is available in Additional file 1.

One of the main strengths of SynTView lies in its highly interactive graphical user interface, possible due to the Flash technology used to develop the software. This technology is increasingly exploited by comparative genomics applications [21,36-38]. It allows the development of rich Internet (or client) applications, which escape the limitations faced by standard HTML websites. Flash also provides advantages similar to or greater than those of other technologies (*e*.*g*., JavaScript and SVG): the Flash plug-in is installed out-of-the-box on most computers and it is fully portable across different web browsers. Although HTML5 is becoming more and more popular on the world-wide web, including for genomics applications (*e*.*g*., [39]), it is currently less cross-compatible among web browsers than Flash and mostly limited to playing videos and animations, rather than rich Internet applications; indeed, development environments and resources are still far more efficient when using Flash/Flex (*e*.*g*., the object serialisation protocol AMF3 in ActionScript/MXML) than using HTML5. In addition, the Flash strategy allows us to propose a web access and a client application (AIR environment) simultaneously. Thus, we have created a versatile solution that offers the best of both worlds: a desktop program that can easily be installed by a non-expert user, providing direct access to his local storage and file systems, without needing an IT infrastructure to deploy complex web architectures; but also, a browser application that may be more intuitive for some users and allows several people to access a single instance of the software, while retaining many of the characteristics of desktop-like programs.

Note that SynTView is not intended for data generation: the software is focused on the dynamic exploration of genome data produced externally. In particular, SynTView is not linked to any particular database, which allows the generation of plain text files that the application will interpret and subsequently represent (thus easily integrating the user’s own data) or the exploitation of other sources of information, such as the GenoList environment.

SynTView was designed to be extremely fast, and sports a user-friendly interface that is straightforward to use for biologists accustomed to graphical widgets. In particular, data exploration is facilitated by the possibility of connecting several views: indeed, in both the synteny and SNP visualisations, the various representations are interactively linked together, and a user selection (*e*.*g*., genes, genome region) in one panel dynamically triggers either a related selection or a view modification in other panels (Figure 1). This is a major feature that is very helpful for making sense out of large and complex data sets, in a way that is not available in most similar software, even if individual analogous views can be found (see below). For example, interesting areas of the chromosome may be observed (*e*.*g*., recombination, inversion) using the line plot representation; it is possible to zoom in on these regions, then select the corresponding BDBH to export them, or shift with one mouse click to the local view to analyse the region in more detail. The export functionalities offered by SynTView are also appropriate for further analysis, once an initial exploration has identified the data sets to be selected. The images generated by the software can be saved in PNG format and subsequently used in reports or publications.

Numerous applications are already available for visualising comparative genomics data from microbial species, but we believe that SynTView fills a gap and is more complete: it is a flexible software package, compatible with various computing environments, and uniquely integrates complementary features present, in isolation, in similar tools. For instance, the CGAT Java alignment viewer [28] and the GSV web package [23] are efficient for syntenic visualisations, but are restricted to pairwise genome comparisons. Likewise, MizBee [19] implements an innovative multi-scale synteny browser that enables interactive exploration of various kinds of relationships, but only between two multi-chromosome genomes at a time. The Cinteny [25] and Genomicus [18] web sites are primarily designed for metazoa; Genomicus focuses on ancestral genome reconstruction and Cinteny provides on-the-fly computed, but non-interactive, synteny views. CGView [14] and Easyfig [26] are highly customisable packages for producing publication-quality, but static-only, figures; the GView [22] Java application is more interactive but mainly focused on the display style configuration. Companion tools to the popular genome browser GBrowse, such as SynBrowse [20] and SynView [29], also offer interactivity albeit limited. Circos [17] and BRIG [7], available as desktop applications, are mostly restricted to static (circular) views. GenomeRing [15] has an interactive web interface for visualising multiple circular genome alignments, based on an original concept of a common annotation coordinate system. Gobe [21] is a web-based Flash application, hence very dynamic, but it is limited to synteny information. Although the Mauve [11] and VISTA [13] packages come with useful Java visualisation interfaces, they are primarily dedicated to whole genome alignments. The MGV [16] and Sybil [24] suites are feature-rich web-only applications for the interactive visualisation of microbial genomic contexts, but these software do not include nucleotide polymorphism information. More recent software were developed especially to handle NGS data sets, although most do not focus on the representation of processed biodiversity information for large collections of microbial strains. They include: IGV [27] and Savant [12] which are both widely used; and GenomeView [6] which is a feature-rich stand-alone genome browser able to cope with dozens of “NGS” genomes, but which lacks integrated circular representations. The latest version of Artemis [8], an established annotation tool, in association with ACT [10] and DNAPlotter [9], provides the user with a large set of comparative views, some of them being dedicated to bacterial population genomics studies. Together, these three Java applications probably come closest to matching the features provided by SynTView.

**Figure 2:**
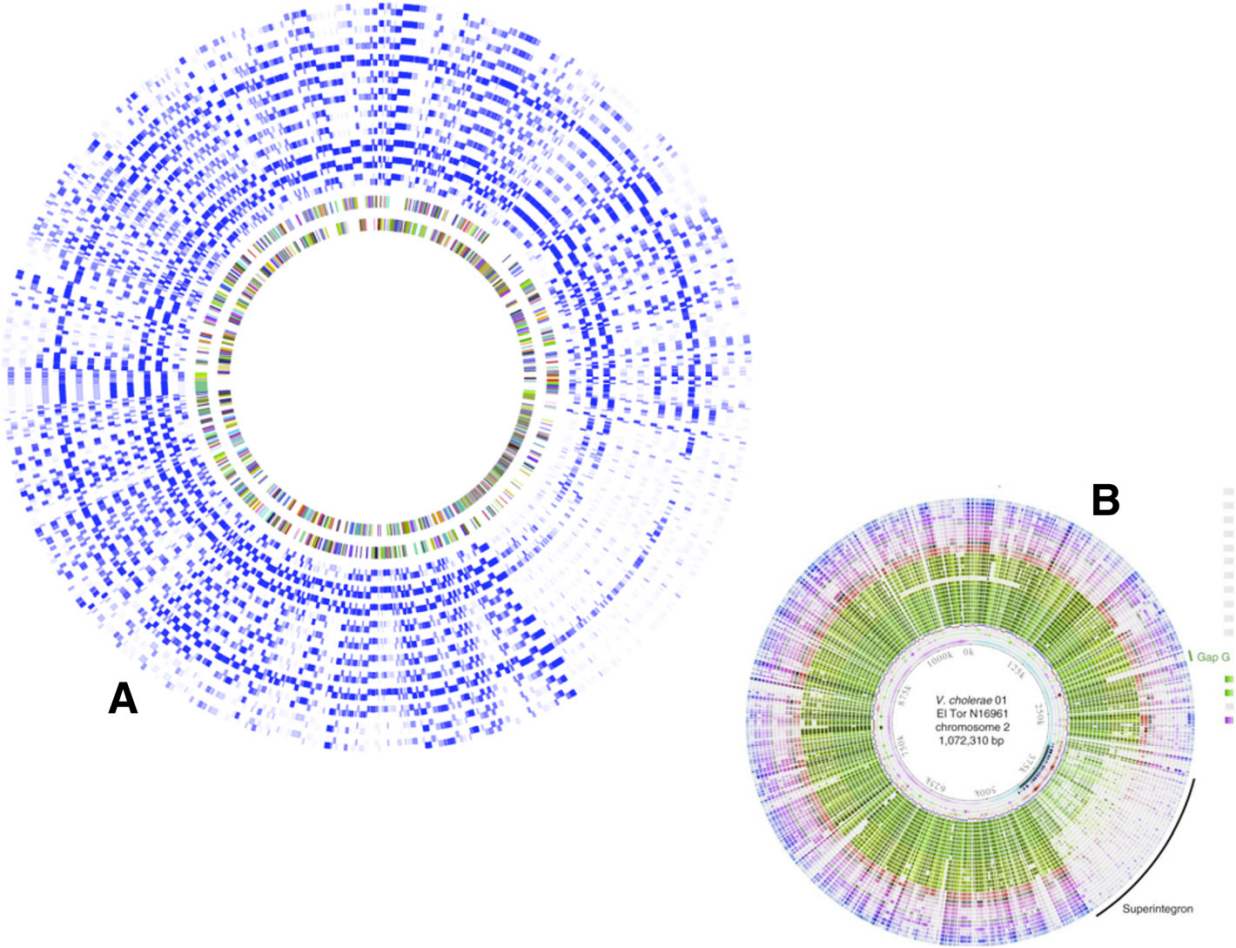
**Identification of particular genome regions through heat map representation. A**: Circular representation of 12 *Vibrionaceae* genomes obtained using SynTView, showing their gene similarity relationships (heat map) and highlighting a particularly sparse region (bottom right). **B**: Figure taken from reference [40] representing the same genomes and describing the specific region highlighted as the superintegron of *Vibrio cholerae*.

**Figure 3:**
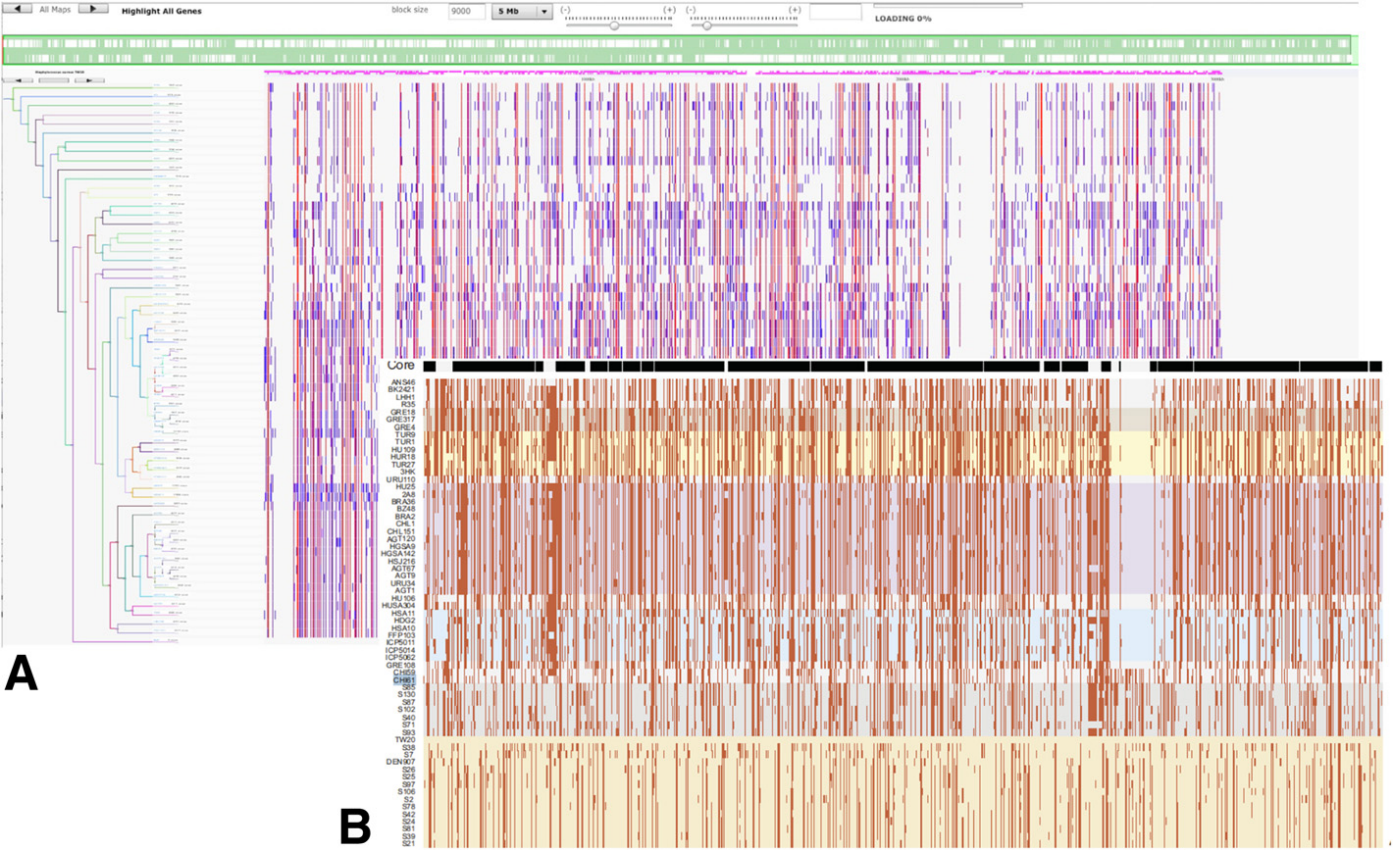
**Exploration of bacterial population genomics data. A**: SynTView representation of SNP heat maps along 62 genomes of *Staphylococcus aureus* ordered using a dynamic phylogenetic tree; the colours represent the frequency of SNP occurrence among the strains (from specific to one strain, to shared by all strains). **B**: Similar representation using the same data, reproduced from reference [41].

SynTView has been used in several genome sequencing projects. Its data visualisation features help to reveal or confirm biological models. For instance, it allowed us to reproduce an observation regarding bacterial genome organisation: the location of the superintegron in *Vibrio* species [40] (Figure 2). SynTView was also used to analyse hundreds of bacterial strains that were Illumina-sequenced and whose nucleotide polymorphism was determined, thus yielding SNP heat map representations similar to those in [41] (Figure 3). Other applications include the analysis of genomic islands visualised by the sequencing coverage representation, allowing confirmation, or otherwise, of the presence or absence of such islands, and the identification of specific deletions therein.

SynTView will be further developed in several ways. First, we plan to increase the scope of the software by accommodating other types of data. In particular, SynTView should enable the management of incomplete genomes for synteny representations. For NGS, we also intend to design novel views for RNA-seq data (such as expression levels or transcription start sites) and to improve the SNP representation such that it can handle multiploid organisms. As far as end-user interactions are concerned, improvements will include features such as the ability to save selected views and gene baskets in a permanent way.

## Conclusions

We have developed software for the dynamic visualisation of microbial genomes, such that both synteny and DNA polymorphism can be displayed. The most important asset of SynTView is its interactivity, inherent to the use of the Flash technology. In addition, several properties of the application facilitate efficient investigation of large data sets at the chromosome scale: for instance, its flexibility for establishing links with various user data sets and the dynamic interactions between the numerous specialised views. This innovative approach to data exploration is not possible with most existing applications, because they are more static and/or do not offer multiple views of multiple genomes.

## Availability and requirements

SynTView is available under the GNU LGPL (Lesser General Public License) 3.0 license at the following URL: <http://genopole.pasteur.fr/SynTView>. As the Flash runtime is pre-installed in most web browsers, the application can run without any further installation. Technical documentation, tutorials and demonstration sites are available at the following URL: <http://genopole.pasteur.fr/SynTView/documentation>.

**Project name**: SynTView

**Project home page**: http://genopole.pasteur.fr/SynTView

**Operating system**(**s**): Platform independent

**Programming language**: ActionScript 3

**Other requirements**: Adobe® Flash Player

**License**: GNU LGPL 3.0

**Any restrictions to use by non**-**academics**: none

## Competing interests

The authors declare that they have no competing interests.

## Authors’ contributions

All authors participated in the design and testing of the application. PL also carried out the following tasks: implementation, documentation, and manuscript drafting. IM wrote the final version of the manuscript. All authors read and approved the final manuscript.

## Supplementary Material

Additional file 1 **Full list of SynTView features.** This file provides an exhaustive description of the functionalities found in the SynTView software. Click here for file
