# Supplementary material for: SynTView — an interactive multi-view genome browser for next-generation comparative microorganism genomics
Source: BMC Bioinformatics. 2013 Sep 22;14:277. doi: 10.1186/1471-2105-14-277 (PMC3849071; doi:10.1186/1471-2105-14-277)
Supplement: Additional file 1 — Full list of SynTView features. This file provides an exhaustive description of the functionalities found in the SynTView software. [file 1471-2105-14-277-S1.doc]

# Additional file 1. Full list of SynTView features.

# Main Global Actions

*Functionalities that are relevant to all graphical components*

*Full Screen*

- Makes the application take the entire screen area

*Gene List*

- Table of the whole set of genes of the pivot replicon, with the following columns: Accession Number|Gene Name|Description|From|To|Direction|Rank|SNPs|Indels|inBasket.
- Sub-table with BDBH genes for each gene of the pivot replicon against comparative genomes

*Actions*

- Connection with the local view centered around the selected gene (with or without synteny display)
- Add genes in the basket (checkbox or upload of a list of accession numbers)
- Sortable columns
- Data export

*Select Genomes*

- Table of genomes with number of genes and size of associated replicons

*Actions*

- Choice of genomes or SNP sub-maps
- (de)Selection of maps to display (in progress)

*Print*

- For each tab, export of the current view in PNG format

# Local View

*Main linear view of a genome region with local synteny or SNP sub-maps*

- Slider for direct access to a genome region of the pivot replicon
- Pan / zoom in the genome
- Representation scale adjustable (25 kb by default)
- Genome stacks: piling up of genomic maps enabling the representation of gene relationships
- Set map ordering by drag and drop
- Set map ordering according to a phylogenetic tree (Newick format) with flippable branches
- Map deletion
- Add metadata about strains to classify and order them
- Gene search by auto-completion on the biological gene name
- Gene drawing
- Gene representation at their genomic coordinates
- Annotation tooltip
- Contextual menu for operating on the gene of interest
  - - Center the representation around this gene
    - Show synteny with links between genes
    - Contextual menu on synteny links
      - Get the related BDBH gene list
      - Get the protein sequences of BDBH genes
      - Computation of the multiple alignment of BDBH sequences (Muscle)
    - Link to cross-references (URL defined in flashVars)
    - Add gene in the basket
    - Highlight the gene in a circular genome view
- Colorization according to the BDBH score
- Animation of SNPs located inside the gene
- SNP drawing
- Display filtering and specific color according to the SNP type (four types of SNPs: intergenic / synonymous / non synonymous / small indel – ≤ 3 nucleotides)
- Different scales/representation modes: (i) individual SNPs colored according to their location with respect to genes, (ii) SNP clusters, (iii) SNP heat maps
- Animation to blink the cognate gene
- Histogram of the sequencing coverage with different representation modes to take into account over-coverage areas
- Gene sequence visualization
- Sequence browser with global and local views
- Possible effects of SNPs on the sequence (nucleotide and amino-acid levels)
- Sequence export
- Artificial sequence visualization
- From whole genomes
- From basket genes (without intergenic SNPs)
- From selected genome regions
- Comparison of the SNP density according to groups of strains
- Autosynteny mode (enabled or not)

# Line Plot

*All possible pairwise genome comparisons in a superimposed arrangement*

- Pan / scale
- Colorization of the region corresponding to the local view, including a clickable link toward this view

# Dot Plot

*All possible pairwise genome comparisons in an orthogonal frame*

- Graphical selection of BDBHs to generate an association table or copy/export the data
- Display filtering according to the annotations
- Return to the local view centered around the selected gene

# Phylogenetic Profile

*Heat map of the pivot replicon against other genomes ordered according to a phylogenetic tree*

- Computed either from the whole genome or from a set of genes
- Box color and shape in relation with the BDBH strength and protein coverage, respectively

# Pan / Core Genome

*Representation of successive core-genomes*

- Genomes added following the local view order to progressively compute core genomes
- BDBH sub-list export (possible at each step of the core genome computation)

# Circular View

*Circular view of the pivot genome*

- Selection line
- Provides the location in kb
- Enables to go to the corresponding location in the local view
- Linked to a synchronized linear mini-map of the surrounding region
- Synteny
- Concentric circles with genes of other genomes (ordered using the track order of the local view)
- Only genes involved in synteny relationships in the local view are colored
- Heat map
- Concentric circles of blocks, the color intensity of which correspond to the strength of the BDBH relationship
- Customizable color gradients
- SNP maps
- Concentric circles with color codes according to the types of SNPs
- Display of the sequencing coverage
- Histogram of the number of SNPs per gene
- Heat map to represent the number of strains sharing a given SNP
- Linked linear mini-map of the pivot replicon
